# Successful chimney endovascular aortic repair with reconstruction of three visceral branches for huge saccular juxtarenal abdominal aortic aneurysm after trans-thoracoabdominal esophagectomy

**DOI:** 10.1186/s13019-024-02784-x

**Published:** 2024-05-04

**Authors:** Takasumi Goto, Hironobu Fujimura, Takashi Shintani, Takashi Shibuya

**Affiliations:** 1 Department of Cardiovascular Surgery, Toyonaka Municipal Hospital, 4-14-1, Shibahara, Toyonaka, Osaka 560-8565 Japan; 2 Department of Cardiovascular Surgery, Osaka University Graduate School of Medicine, Suita, Japan

**Keywords:** Chimney endovascular aortic repair, Juxtarenal abdominal aortic aneurysm, Saccular aneurysm

## Abstract

**Background:**

Conventional graft replacement for a juxtarenal abdominal aortic aneurysm (JRAAA) remains challenging for high-risk patients since it often requires the reconstruction of some visceral arteries.

**Case Presentation:**

A 76-year-old woman was diagnosed with an 87 × 48 mm saccular JRAAA. Open graft replacement was contraindicated because of frailty and a past history of trans-thoracoabdominal esophagectomy. Chimney endovascular aortic repair (ChEVAR) with three chimney endografts was successfully performed without any endoleaks, and each visceral circulation was kept intact. The patient was discharged uneventfully on postoperative day 8. Significant shrinkage of the aneurysmal sac and preservation of flow through each chimney graft were observed on computed tomography 6 months postoperatively, with no significant increase in serum creatinine levels on laboratory testing.

**Conclusions:**

ChEVAR can be a useful surgical option instead of conventional operations, especially for high-risk cases.

**Supplementary Information:**

The online version contains supplementary material available at 10.1186/s13019-024-02784-x.

## Background

For a juxtarenal abdominal aortic aneurysm (JRAAA), the first-line surgical treatment is open graft replacement (GR), which usually requires the reconstruction of the renal artery (RA) or superior mesenteric artery (SMA). The mortality and morbidity rates for JRAAA are higher than those following GR for infrarenal AAA [1]. The development of devices and technical improvements in endovascular aortic repair (EVAR) have contributed to extending the proximal landing zone by the reconstruction of the visceral branches, leading to the feasibility of EVAR for some JRAAA [1–4].

We report a successful case of chimney EVAR (ChEVAR) for a huge saccular JRAAA after trans-thoracoabdominal esophagectomy.

## Case presentation

**Fig. 1:**
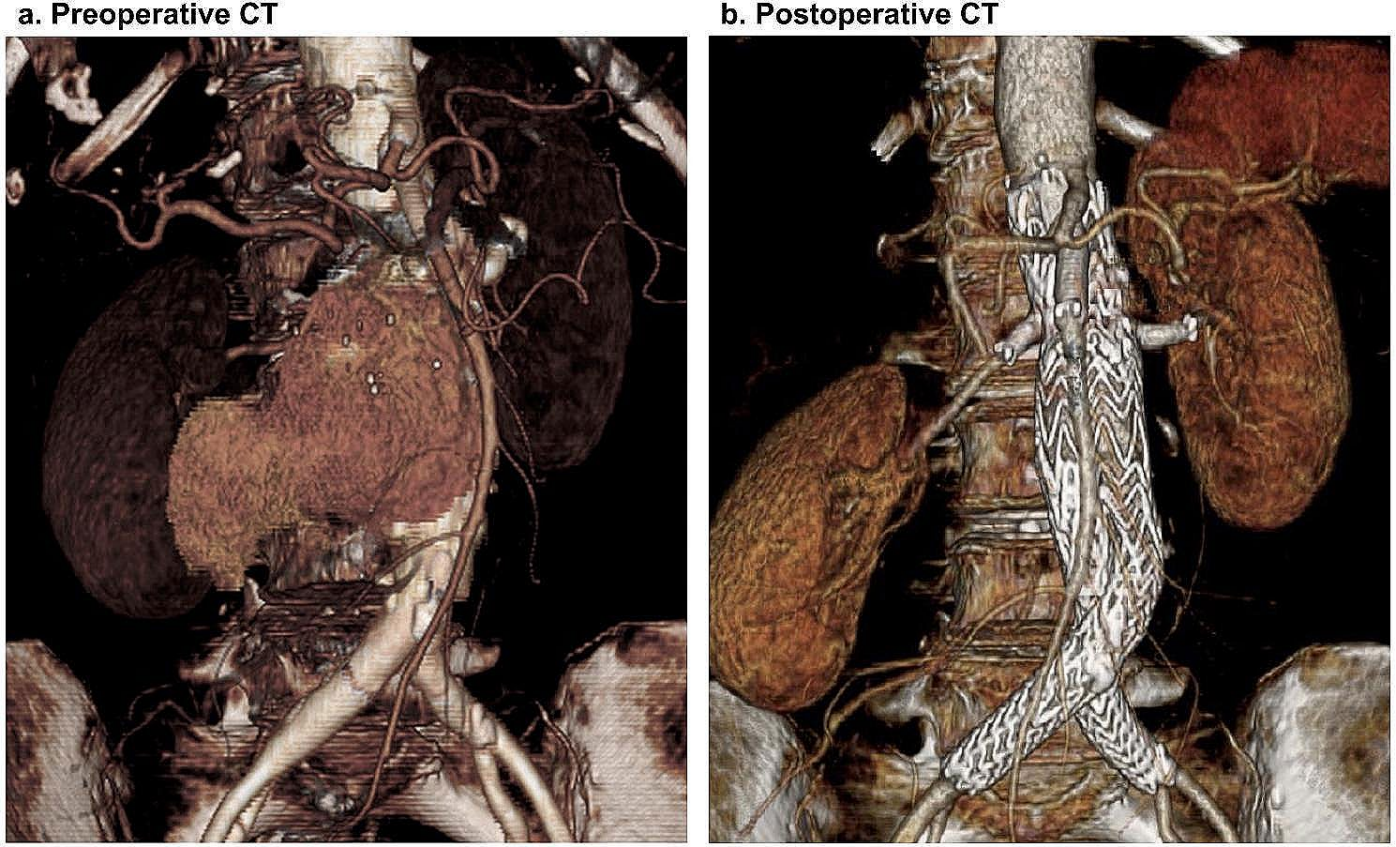
Preoperative and postoperative CT findings **a**: Preoperative CT shows that the juxtarenal abdominal aortic aneurysm is enlarged toward the right side. **b**: ChEVAR was successfully performed with preservation of visceral circulation ChEVAR, chimney endovascular aortic repair; CT, computed tomography

**Fig. 2:**
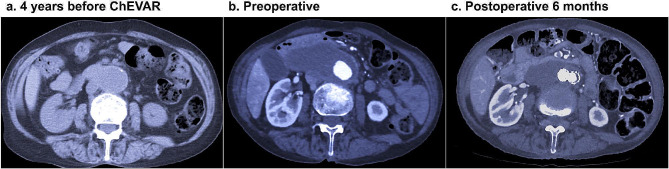
Change in size of the saccular JRAAA from 4 years preoperatively to 6 months postoperatively. **a**: The axial view of JRAA at 4 years before ChEVAR, measuring 54.1 × 32.3 mm in size. **b**: Preoperative CT findings clarified significant enlargement of JRAAA toward the sac wall side. **c**: CT 6 months postoperatively demonstrates noticeable reduction of JRAAA. ChEVAR, chimney endovascular aortic repair; CT, computed tomography; JRAAA, juxtarenal abdominal aortic aneurysm

A 76-year-old woman, who had previously undergone trans-thoracoabdominal esophagectomy with gastric tube reconstruction, was admitted for aspiration pneumonia. An impending rupture of AAA was suspected based on enhanced computed tomography (CT) findings; therefore, she was referred to our department. The AAA was located 5 mm below the left RA, with the aetiology being a saccular degenerative aneurysm. The AAA was enlarged exclusively towards the right side, measuring 87 × 39 mm (Figs. 1 and 2). There were no indications of infection surrounding the aneurysm, especially around the sac. No signs associated with AAA rupture were present. Thus, the patient was diagnosed with a saccular JRAAA. Regarding the initial laboratory data, systemic inflammatory markers were significantly increased by aspiration pneumonia (Additional file 1). The blood culture tests were conducted several times, and all these results were negative. Prior to surgical treatment for JRAAA, appropriate antibiotic therapy (Ampicillin-Sulbactam 3.0 g x 4 times/day) was provided for 10 days, and the pneumonia and following systemic inflammatory reactions had improved consequently.

Given her relatively young age, GR was initially planned. However, the operative risk was estimated to be high because of past trans-thoracoabdominal esophagectomy 40 years previously and her frail condition unrelated to the cancer, with a body weight of 35 kg. Anatomical findings showed that the diameter of the thoracoabdominal aorta between the celiac artery (CA) and RA ranged from 21 to 23 mm, with over 50 mm available for the proximal landing zone (Additional file 2). Regarding the diameter of each visceral branch, that of the SMA, right RA, and left RA measured 6.0, 4.5 and 4.2 mm in size, respectively. In 1-ChEVAR with reconstruction of the left RA, the length of the proximal landing zone was not enough long. Using 2-ChEVAR with reconstruction of the bilateral RAs would achieve a longer proximal landing zone than 1-ChEVAR. The length from the top of the right RA orifice to the bottom of the SMA orifice was about 10 mm. We decided this length might be insufficient to prevent gutter endoleak. We also considered that 2-ChEVAR with reconstruction of bilateral RAs would not be suitable because of the risk for potential accidental occlusion of the SMA orifice by those two chimney grafts. Thus, we planned to perform ChEVAR with three chimney endografts.

**Fig. 3:**
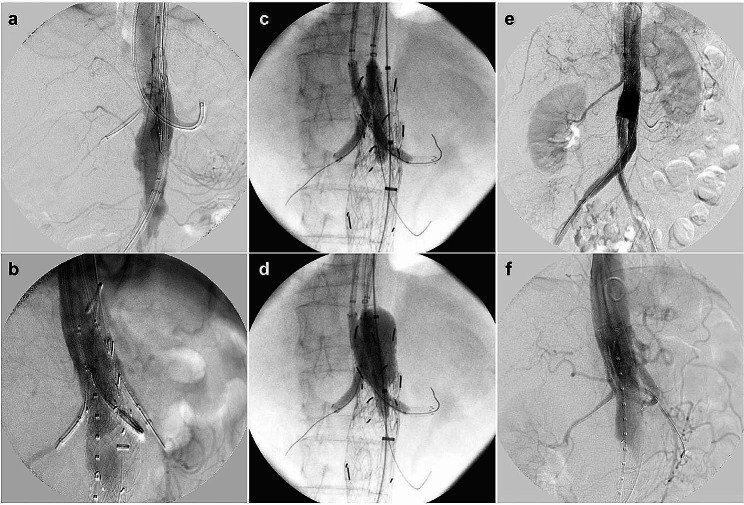
Intraoperative angiography findings. **a, b**: Front and RAO 50° view after sheath cannulation into each visceral branch. **c, d**: Balloon touch up for each chimney graft and main body. **e, f**: Final aortography shows no endoleaks and preservation of each visceral circulation (front and RAO 50° view)

After general anaesthesia, 7-Fr long sheaths were inserted into the SMA and bilateral RAs through the exposed bilateral axillary arteries (Fig. 3a and b). A 7-mm endograft (VIABAHN, W.L. Gore and Associates, Flagstaff, AZ) and two 5-mm endografts (VIABAHN) were inserted into the SMA and bilateral RAs, respectively, along a 0.018-inch wire (V-18™ control wire, Boston Scientific) via each sheath. Subsequently, EVAR was performed by using a 26-mm stent graft (Excluder 26-12-140, W.L. Gore and Associates, Inc., USA) and contralateral leg endograft (Excluder 12–100) through the exposed common femoral arteries, using Dry seal sheaths (W.L. Gore and Associates) sized 16- and 12-Fr, respectively. The proximal extension device (aorta extension 28.5–33, W.L. Gore and Associates) was deployed from under the CA. After the deployment of all chimney endografts in the appropriate positions, ballooning to the main device and chimney endografts was performed simultaneously (Fig. 3c and d). The aortography showed no endoleaks (Fig. 3e and f). The total operative time was 259 min (Additional file 3). The patient was discharged uneventfully on postoperative day 8. Postoperative CT showed preservation of visceral circulation, without endoleaks (Figs. 1b and 2b). The follow-up CT at 6 months postoperatively showed significant shrinkage of the aneurysmal sac and preservation of visceral branch flow (Fig. 2c). Laboratory tests showed no deterioration of renal function and no elevation of systemic inflammation markers (Additional file 4, 5).

## Discussion and conclusions

In this case, severe adhesions due to past trans-thoracoabdominal esophagectomy were anticipated, and the aorta beside the saccular aneurysm could be fragile. The risk of perioperative rupture was estimated to be high. Additionally, the potential risk of anastomotic pseudoaneurysm could be higher than that of usual AAA owing to the fragility of the aorta. In such a situation, EVAR is better indicated than GR since surgical suture to the aorta is not required [1, 5, 6]. As for fenestrated EVAR, including the use of physician-modified endografts, the early outcomes are satisfactory [1–4]. However, it requires an order-made device, which can take four weeks to prepare. The use of physician-modified endografts would be difficult for many physicians since specific methods are required for preparing the graft. Therefore, it was unsuitable for this patient since she had a huge saccular aneurysm that would have enlarged rapidly in a short period of time (Fig. 2).

With respect to the aetiology, mycotic aneurysm would be one of the differential aetiologies to consider. Endovascular therapy is unsuitable for those who have such a background since it cannot control the localised infection. In the present case, preoperative enhanced CT findings demonstrated no evidence of local infection, such as strong enhancement around the sac. Increased levels of inflammatory markers on admission suggested a systemic reaction for aspiration pneumonia. The blood culture results were negative, and serum β-D glucan level was lower than the cut-off value (Additional file 1). Given those CT and laboratory test findings preoperatively, this case seemed to be an advancement of saccular degenerative AAA and not of mycotic aneurysm.

A type-Ia endoleak is a serious complication after ChEVAR [1, 2], which theoretically could be avoided by obtaining sufficient proximal landing length. In this case, the length between the aneurysm neck and SMA was 33 mm, and that between the aneurysm neck and CA was 53 mm. Based on these findings, we planned ChEVAR with three chimney endografts. Ultimately, postoperative CT demonstrated no endoleaks, and the visceral circulation was preserved (Fig. 2b, Additional file 5a).

The long-term patency of the chimney endograft remains uncertain, with the reported patency at one and three years postoperatively being 94% and 87%, respectively [1, 2]. To evaluate the patency of the chimney graft and the durability of ChEVAR, we plan to continue periodical follow-up CT in the future, specifically at least every 3 months during the first year. At 6 months postoperatively, there were no signs of obstruction of the chimney grafts (Additional file 5b), and a significant decrease in the sac diameter was found (Fig. 2c). For further assessments of the durability, periodical follow-up CT should be performed over a longer period.

In conclusion, ChEVAR is an attractive surgical option, particularly for high-risk cases.

### Electronic supplementary material

Below is the link to the electronic supplementary material.

Additional file 1: Details of the laboratory test on admission

Additional file 2: ChEVAR planning sketch

Additional file 3: Intraoperative movie

Additional file 4: Details of the laboratory test on admission

Additional file 5: Axial CT images of 3 chimney grafts

## Data Availability

All data generated during this study are included in this published article.

## Abbreviations

JRAAA: Juxtarenal abdominal aortic aneurysm
GR: Graft replacement
RA: Renal artery
SMA: Superior mesenteric artery
EVAR: Endovascular aortic repair
ChEVAR: Chimney endovascular aortic repair
CT: computed tomography
CA: Celiac artery
