# Supplementary figures and images for: Successful chimney endovascular aortic repair with reconstruction of three visceral branches for huge saccular juxtarenal abdominal aortic aneurysm after trans-thoracoabdominal esophagectomy
Source: J Cardiothorac Surg. 2024 May 4;19:276. doi: 10.1186/s13019-024-02784-x (PMC11071143; doi:10.1186/s13019-024-02784-x)

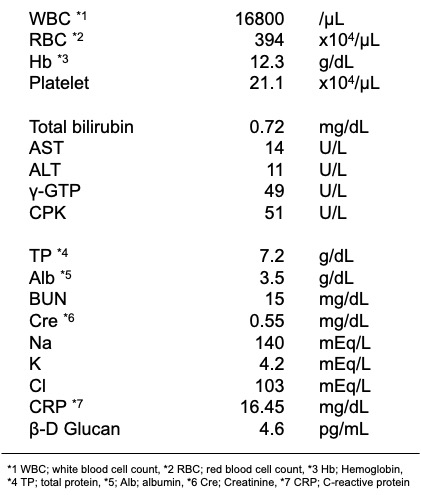

Supplement: Supplementary file 1 — Additional file 1: Details of the laboratory test on admission [file 13019_2024_2784_MOESM1_ESM.jpg]

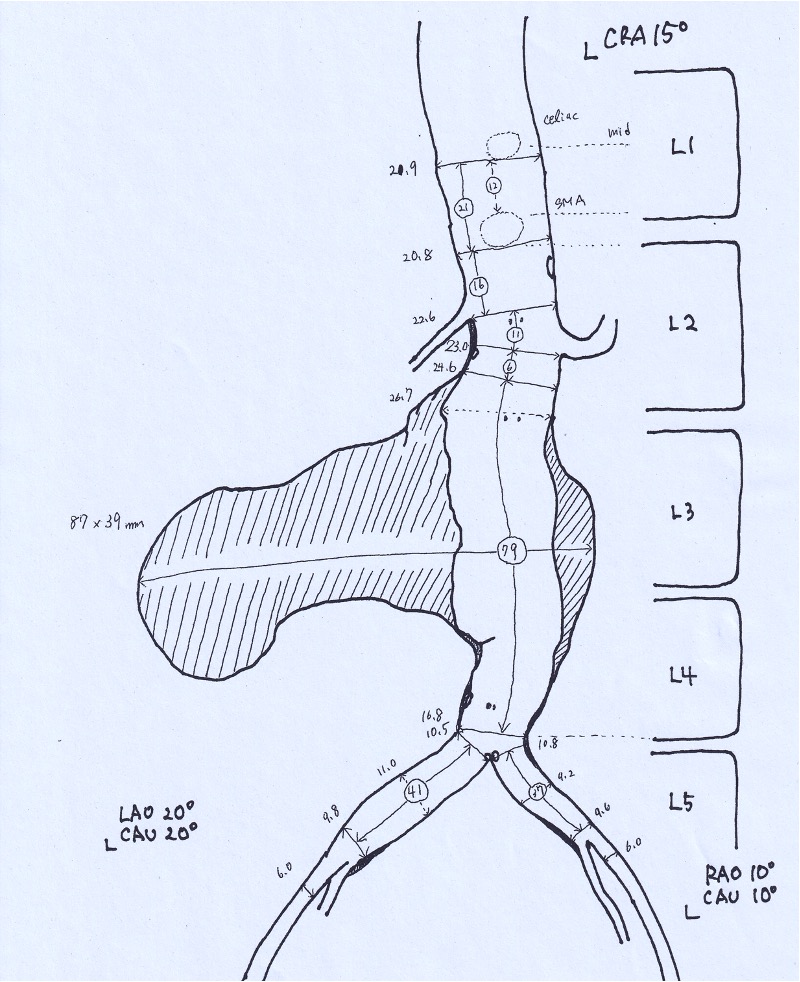

Supplement: Supplementary file 2 — Additional file 2: ChEVAR planning sketch [file 13019_2024_2784_MOESM2_ESM.jpg]

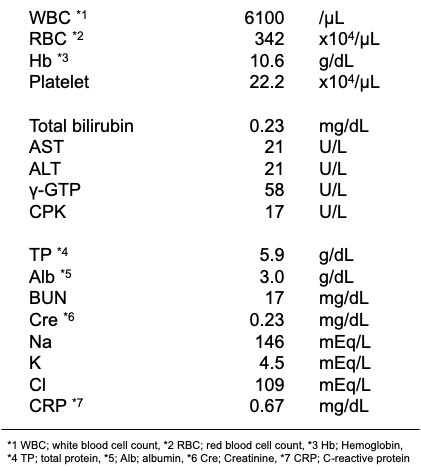

Supplement: Supplementary file 3 — Additional file 3: Intraoperative movie [file 13019_2024_2784_MOESM3_ESM.jpg]

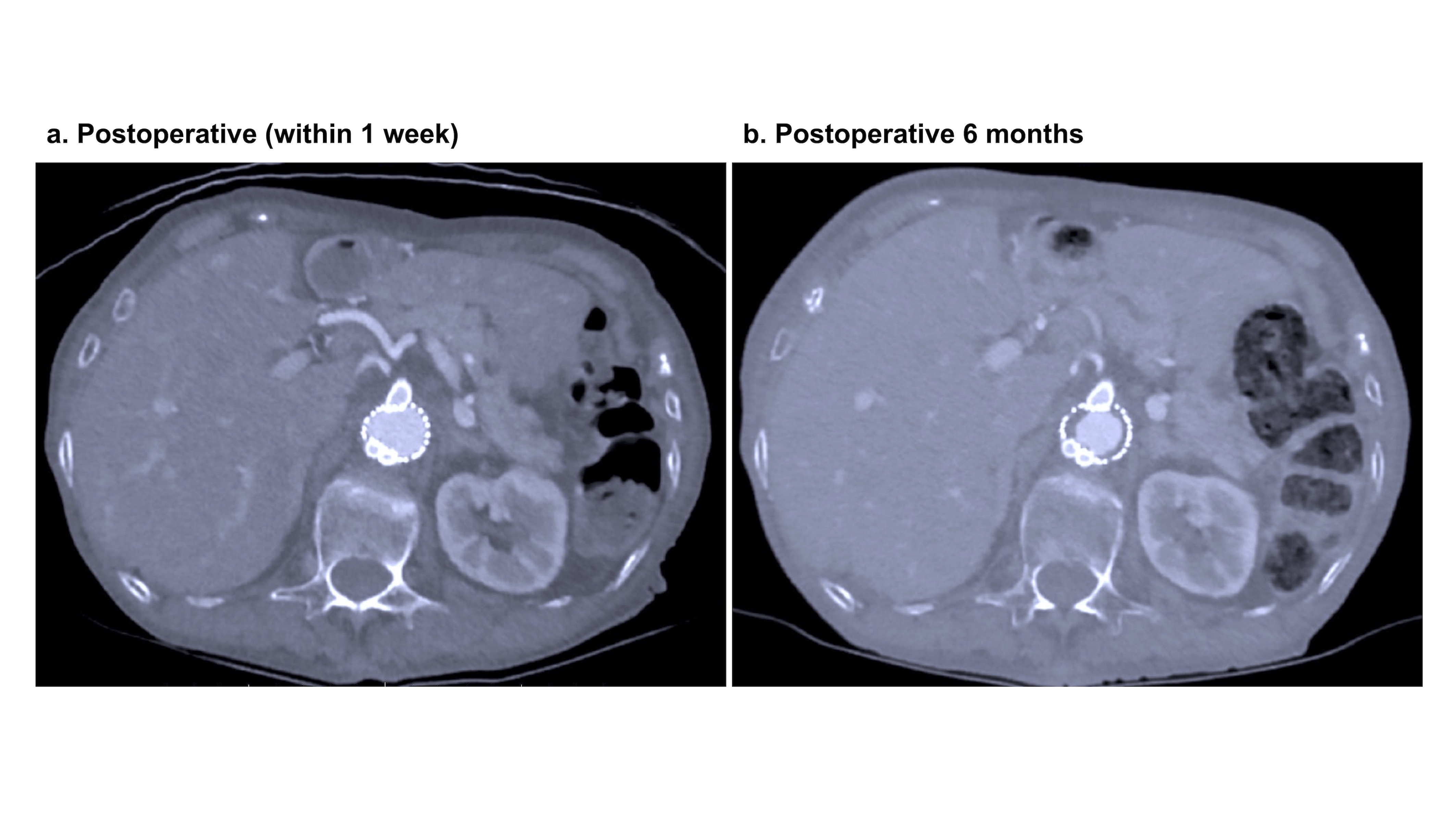

Supplement: Supplementary file 5 — Additional file 5: Axial CT images of 3 chimney grafts [file 13019_2024_2784_MOESM5_ESM.jpg]
